# Chasing the structural diversity of the transcription regulator *Mycobacterium tuberculosis* HigA2

**DOI:** 10.1107/S2052252521007715

**Published:** 2021-08-24

**Authors:** William Richardson, Gyun Won Kang, Hee Joong Lee, Kang Mu Kwon, Saron Kim, Hyo Jung Kim

**Affiliations:** a Centre for Biomolecular Sciences, School of Pharmacy, University of Nottingham, Nottingham, United Kingdom; b College of Pharmacy, Woosuk University, Wanju 55338, Republic of Korea

**Keywords:** toxin–antitoxin systems, *Mycobacterium tuberculosis*, transcription factors, HigBA, HigA2, crystal structure, structure determination, X-ray crystallography

## Abstract

Transcription factors are the primary regulators of gene expression and recognize specific DNA sequences under diverse physiological conditions. Although they are vital for many important cellular processes, it remains unclear when and how transcription factors and DNA interact. The antitoxin from a toxin–antitoxin system is an example of negative transcriptional autoregulation: during expression of the cognate toxin it is suppressed through binding to a specific DNA sequence. In the present study, the antitoxin HigA2 from *Mycobacterium tuberculosis* M37Rv was structurally examined. The crystal structure of *M. tuberculosis* HigA2 comprises three sections: an N-terminal autocleavage region, an α-helix bundle which contains an HTH motif, and a C-terminal β-lid. The N-terminal region is responsible for toxin binding, but was shown to cleave spontaneously in its absence. The HTH motif performs a key role in DNA binding, with the C-terminal β-lid influencing the interaction by mediating the distance between the motifs. However, *M. tuberculosis* HigA2 exhibits a unique coordination of the HTH motif and no DNA-binding activity is detected. Three crystal structures of *M. tuberculosis* HigA2 show a flexible alignment of the HTH motif, which implies that the motif undergoes structural rearrangement to interact with DNA. This study reveals the molecular mechanisms of how transcription factors interact with partner proteins or DNA.

## Abstract 2

Three crystal structures of the transcription regulator HigA2 from *Mycobacterium tuberculosis* reveal a flexible dimerization mode, which affects its interaction with DNA.

## 1. Introduction   

Bacterial gene regulation is controlled by a multitude of transcription factors that recognize specific DNA sequences and allow controlled cellular responses (Villard, 2004 ▸). Given their role in responding to environmental cues such as host colonization and virulence, transcription factors have been intensively studied as drug targets (Liu *et al.*, 2015 ▸). Most transcription factors are multidomain proteins that possess a DNA-binding domain and an effector domain for ligand or protein interaction. There are many known structural motifs for DNA binding, including helix–turn–helix, zinc-finger, leucine-zipper and helix–loop–helix motifs. The simplest motif, helix–turn–helix (HTH), comprises two α-helices with a fixed angle permitting binding to the major groove of DNA (Luscombe *et al.*, 2000 ▸). Since the amino-acid side chains of α-helices are solvent-accessible, they invariably make key interactions with DNA, with their composition influencing the specificity. HTH motif-containing proteins typically form dimers that strengthen their DNA interaction and can mirror the dyad symmetry of their binding site (Brennan & Matthews, 1989 ▸).

In the present study, we elucidated the crystal structure of *Mycobacterium tuberculosis* H37Rv HigA2 (hereafter referred to as _Mt_HigA2), which contains an HTH motif (Sala *et al.*, 2014 ▸). HigB and HigA constitute a bacterial toxin–antitoxin (TA) system which is organized into a small operon. The HigA antitoxin represses the transcription of HigBA or forms a stable HigBA complex, thereby preventing the ribonuclease activity of the HigB toxin and the associated cytotoxic events. However, antitoxins are known to be actively degraded, with the liberated toxins leading to increased pathogenicity (Maisonneuve & Gerdes, 2014 ▸; Schureck *et al.*, 2016 ▸). There are five well known TA systems based on their modes of interaction. Toxins bind to either RNA antitoxins (types I and III) or protein antitoxins (types II, IV and V) (Ghafourian *et al.*, 2014 ▸). Type II is well characterized and abundant, and functions by binding of the antitoxin protein to either the DNA or the toxin (Fraikin *et al.*, 2020 ▸). The HigBA system is a type II TA system that is found in many pathogens, including *Pseudomonas aeruginosa*, *Proteus vulgaris*, *Vibrio cholerae*, *Streptococcus pneumoniae* and *M. tuberculosis* (Kędzierska & Hayes, 2016 ▸).

*M. tuberculosis* is the causative organism of tuberculosis and is a significant contributor to global mortality, with the World Health Organization reporting 1.4 million deaths in 2019 (Fukunaga *et al.*, 2021 ▸). *M. tuberculosis* usually colonizes the lungs and can persist in host tissues for decades without leading to disease, and spreads easily through air transmission. Current treatment regimens are lengthy and consist of a 6–9 month course of four antibiotics, with some concern regarding rifampicin-resistant and multidrug-resistant (MDR) *M. tuberculosis* (Mabhula & Singh, 2019 ▸). Little is known about the inter­actions between the host and bacteria during persistent infection or drug resistance, but it has been suggested that TA systems play an essential role. The genome of pathogenic *M. tuberculosis* H37Rv has 79 TA systems, while other nonpathogenic mycobacterial genomes possess only 5–10 TA systems (Sala *et al.*, 2014 ▸). The risk of active tuberculosis infection increases when the toxin is released from the antitoxin (Gupta, 2009 ▸; Ramage *et al.*, 2009 ▸). Among the 79 TA systems in *M. tuberculosis* H37Rv, there are 38 type II TA systems, which include two HigBA systems: _Mt_HigBA2 and _Mt_HigBA3. Of the two, _Mt_HigBA2 is categorized among ten of the 79 TA systems which are induced drastically in drug-tolerant persister cells. In addition, _Mt_HigBA2 is known to be important for survival in lung tissue (Stewart *et al.*, 2005 ▸; Jain *et al.*, 2007 ▸). In this study, we determined the crystal structure of _Mt_HigA2, a transcription factor from the tuberculosis-causing pathogen *M. tuberculosis* H37Rv. _Mt_HigA2 exploits structural characteristics to interact with the toxin or DNA. This study presents a better understanding of how a multifunctional transcription factor regulates its function through structural changes. This work should contribute new insights into pathogenic bacterial physiology and pathogenicity.

## 2. Materials and methods   

### 2.1. Cloning, expression and purification   

The gene encoding the _Mt_HigA2 antitoxin was amplified from *M. tuberculosis* H37Rv genomic DNA by PCR using 5′-CCAGGGAGCAGCCTCGATGGCGATGACACTACGGGGACATGGAC-3′ and 5′-CCAGGGAGCAGCCTCGCTATGCCAGGGTGAATGTCTCATCTCC-3′ as the forward and reverse primers, respectively. A plasmid for _Mt_HigA2 was prepared using a ligation-independent cloning (LIC) strategy based on a locally engineered pET-15b vector as described previously (Aslanidis & de Jong, 1990 ▸; Eschenfeldt *et al.*, 2009 ▸; Jeong *et al.*, 2012 ▸; Kim *et al.*, 2018 ▸; Kim, 2020 ▸). The amplified DNA of _Mt_HigA2 was inserted into an engineered vector containing an additional thioredoxin (Trx) tag linked by a Tobacco etch virus (TEV) protease cleavage site (Parks *et al.*, 1994 ▸). The resulting construct consisted of an N-terminal hexahistidine tag, a TEV cleavage site, GAAS for LIC and the _Mt_HigA2 gene. Each recombinant plasmid was transformed into *Escherichia coli* DH5α cells and verified by DNA sequencing. For expression, the recombinant plasmid was transformed into *E. coli* C41 cells. The cells were grown in Luria broth (LB) medium supplemented with ampicillin (50 µg ml^−1^) at 37°C. Expression of recombinant Trx-_Mt_HigA2 protein was induced by the addition of 0.5 m*M* isopropyl β-d-1-thio­galactopyranoside (IPTG) upon reaching an OD_600_ of 0.5 and the culture was grown at 37°C for an additional 4 h. The cells were harvested by centrifugation at 4500*g* at 4°C. The cell pellet was resuspended and lysed on ice by sonication in lysis buffer *A* (50 m*M* Tris–HCl pH 7.5, 500 m*M* NaCl), and the lysate was clarified by centrifugation at 20 000*g* for 1 h at 4°C. The cleared supernatant was applied onto an Ni^2+^–nitrilo­triacetate (Ni–NTA) affinity column (Qiagen, Germany) and eluted with elution buffer containing 250 m*M* imidazole. To remove the Trx tag, _Mt_HigA2 was incubated with TEV protease at a 10:1 molar ratio of _Mt_HigA2:TEV protease for 1 h at 4°C. Since TEV protease possesses a hexahistidine tag, only cleaved _Mt_HigA2 was retrieved using an Ni–NTA affinity column. The purified protein was analyzed with >95% purity by SDS–PAGE and was concentrated to 8 mg ml^−1^ by ultrafiltration in 3000 Da molecular-mass cutoff spin columns (Millipore, USA).

### 2.2. Crystallization, data collection and structure determination   

Crystals of _Mt_HigA2 were grown by sitting-drop vapour diffusion at 20°C using a 96-well crystallization plate. Initial crystallization conditions were established using screening kits from Hampton Research (Crystal Screen, Crystal Screen 2, Index, PEG/Ion and Natrix), Molecular Dimensions (ProPlex, JCSG-plus and Structure Screen I and II) and Emerald Bio­Systems (Wizard I, II, III and IV). For the optimal growth of _Mt_HigA2 crystals, 1 µl (8 mg ml^−1^) _Mt_HigA2 solution was mixed with 1 µl precipitant solution and equilibrated against a 1 ml reservoir of the precipitant solution. The best crystals of _Mt_HigA2 were obtained using three conditions: (i) 11%(*w*/*v*) PEG 20K, 0.1 *M* MES pH 6.5; (ii) 10%(*w*/*v*) PEG 8K, 8%(*v*/*v*) ethylene glycol, 0.1 *M* HEPES pH 7.5; and (iii) 25%(*w*/*v*) PEG 4K, 0.2 *M* ammonium sulfate, 0.1 *M* sodium acetate trihydrate pH 4.6. Crystals were transferred to a cryoprotectant solution containing 20%(*v*/*v*) glycerol in each crystallization condition and were flash-cooled in a stream of nitrogen at 100 K. Diffraction data were collected on the BL-5C experimental station at Pohang Light Source (PLS), Korea and the I04 experimental station at Diamond Light Source (DLS), UK. The data sets were processed and scaled using *XDS* and the *CCP*4 suite (Kabsch, 2010 ▸; Winn *et al.*, 2011 ▸). The three crystal forms belonged to space groups *P*2_1_2_1_2_1_, *P*4_3_2_1_2 and *P*3_1_21, respectively. The structure packed in space group *P*2_1_2_1_2_1_ was solved by molecular replacement with *Phaser*, using an ensemble search model generated from PDB entries 1y7y, 3b7h, 3kxa and 2a6c by *MrBUMP* in *CCP*4 (McCoy *et al.*, 2007 ▸; Keegan *et al.*, 2018 ▸; McGeehan *et al.*, 2005 ▸; Ren *et al.*, 2010 ▸). It produced a marginal solution with three monomers in the asymmetric unit with a log-likelihood gain (LLG) of 134. The model was then rebuilt automatically with *ARP*/*wARP* and manually with *Coot*, and refined with *REFMAC*5 and *Phenix* (Emsley *et al.*, 2010 ▸; Liebschner *et al.*, 2019 ▸; Murshudov *et al.*, 2011 ▸; Afonine *et al.*, 2012 ▸; Langer *et al.*, 2008 ▸).

For structural determination of the *P*4_3_2_1_2 and *P*3_1_21 crystal forms, molecular replacement was used in *Phaser* using the _Mt_HigA2 structure packed in space group *P*2_1_2_1_2_1_ as the template. Iterative cycles of model building were performed using *Coot*, followed by refinement in *REFMAC*5 and *Phenix* (Emsley *et al.*, 2010 ▸; Liebschner *et al.*, 2019 ▸; Murshudov *et al.*, 2011 ▸). The crystal contained two dimers (four monomers) per asymmetric unit when the structure was packed in space groups *P*2_1_2_1_2_1_ and *P*4_3_2_1_2. Packing in space group *P*3_1_21 showed one more monomer, with a total of five monomers in the asymmetric unit, and the unpaired monomer forms a crystallographic dimer with twofold symmetry. A portion of the data (5%) were set aside before refinement. The final crystallographic statistics are summarized in Table 1 ▸. Structural alignments and figures were generated using *PyMOL* (http://www.pymol.org) and *UCSF Chimera* (Pettersen *et al.*, 2004 ▸).

### 2.3. PDB codes   

Protein coordinates and structure factors have been deposited in the RCSB PDB as entries 7ewc, 7ewd and 7ewe.

## 3. Results   

### 3.1. The β-lid anchors the dimeric state in the crystal structure of *M. tuberculosis* HigA2   

**Figure 1:**
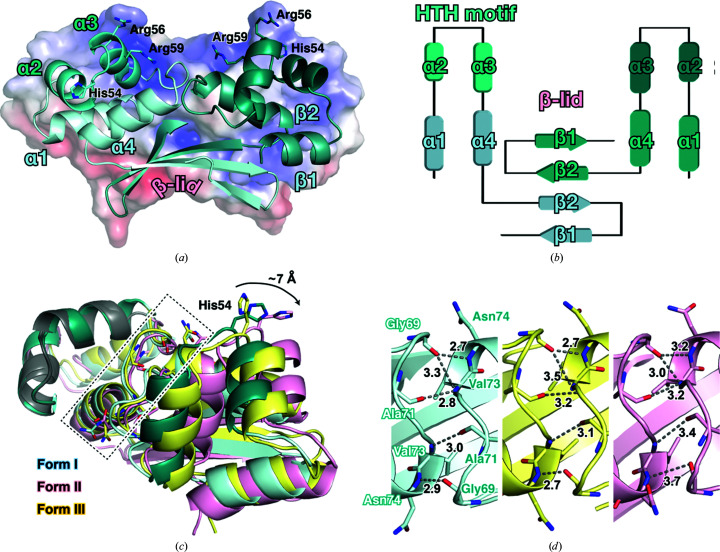
Crystal structure of _Mt_HigA2. (*a*) Crystal structure of the _Mt_HigA2 dimer in ribbon representation. The two monomers are coloured light and dark cyan. The HTH motif is highlighted in darker colours. Three positively charged side chains on the HTH motif are indicated as sticks. Potential surface charge is indicated in the background, where surfaces are coloured between −10 kcal mol^−1^ (red) and +10 kcal mol^−1^ (blue). (*b*) Secondary-structure diagram of the _Mt_HigA2 dimer. The colours correspond to those in (*a*). (*c*) The three forms of the _Mt_HigA2 crystal structure when the HTH motifs are aligned. The arrangement of the second HTH motif is diverse. The side chain of His54 moves ∼7 Å depending on the crystal form. (*d*) Detailed view of the dimerization interface enclosed with a dotted box in (*c*). Hydrogen bonds are indicated as grey dotted lines and distances are given in Å.

The crystal structure of _Mt_HigA2 was determined from three distinct crystal forms (Supplementary Fig. S1). Form I belonged to space group *P*2_1_2_1_2_1_ and was determined to 2.0 Å resolution, form II packed in space group *P*4_3_2_1_2 and was determined to 3.2 Å resolution, and form III was solved in space group *P*3_1_21 to 3.4 Å resolution. Due to a lack of electron density at the N-terminus, ∼25 residues are undefined in each monomer despite the relatively high resolution. The N-terminally cleaved monomer consists of four consecutive α-helices (α1, α2, α3 and α4) and two antiparallel β-strands (β1 and β2), named the α-helix bundle and β-lid after the previously determined HipB antitoxin structure (Schumacher *et al.*, 2009 ▸). The α-helix bundle contains an HTH motif that is required for DNA binding, comprising a preceding helix (α2) and a recognition helix (α3) (Matthews *et al.*, 1982 ▸; Wintjens & Rooman, 1996 ▸). In the _Mt_HigA2 crystal structure, positively charged residues on the HTH motif, His54, Arg56 and Arg59, are oriented towards the expected DNA-binding region. The role of dimerization is delegated to the C-terminal β-lid interface, which comprises two antiparallel β-strands from each monomer that stack to form a four-stranded antiparallel β-sheet, referred to as a β-lid, due to its curvature. A substantial number of β-lid hydrogen bonds and salt bridges result in a tight dimerization network [Figs. 1 ▸(*a*) and 1 ▸(*b*)].

Although dimerization is largely dependent on the β-lid, some limited interaction is observed between the α-helix bundles through the hydrogen bonding of backbone atoms. However, the interaction distances are diverse between the structures determined from the crystal forms. All dimers share the β-lid as their major dimeric interface, but the position of the α-helix bundle varies. When the different dimers are aligned based on a single HTH motif from one monomer, the corresponding motif in the dimer pair shows a different relative position. The flexibility in dimerization leads to drastic changes in the locations of positively charged residues on the HTH motif, including His54, Arg56 and Arg59. The greatest change in distance is that between the N^δ^ atoms of His54, which move by ∼7 Å [Figs. 1 ▸(*c*) and 1 ▸(*d*)]. This implies that flexibility in dimerization would have a huge impact on DNA bending. To predict the physiological dimer, each form was submitted to the *PISA* server to calculate the strength of the dimer interface. Interestingly, all of the forms had similarly favourable dissociation energies (Δ*G*): −14, −15 and −17 kcal mol^−1^ for forms I, II and III, respectively. This suggests that the flexibility of the β-lid does not impact the formation of a stable dimer and is likely to contribute to function.

### 3.2. Autocleavage of *M. tuberculosis* HigA2   

_Mt_HigA2 is a 101-amino-acid protein (11 kDa) that forms a dimeric state in solution. However, the N-terminal ∼25 residues are unstructured in all three observed crystal forms. Interestingly, this agrees with our previous structural observations on _Mt_HigA3, which was also determined to have a cleaved N-terminus. Although crystallization was attempted in the presence of a covalently linked Trx at the N-terminus (∼13 kDa), the whole Trx was not packed in the crystal, implying that the protein tends to be cleaved spontaneously. A similar result was observed for *V. cholerae* HigA, which was crystallized with and without the cognate HigB toxin. The N-terminal ∼25 residues are not structured when crystallized without the toxin. These cleaved residues were found to interact with the toxin through the elucidation of a toxin–antitoxin complex crystal structure (Hadži *et al.*, 2017 ▸). The same pattern is shown in the crystal structure of *S. pneumoniae* HigA, indicating that the missing N-terminus in the _Mt_HigA2 structure is important for interaction with the _Mt_HigB2 toxin (Kang *et al.*, 2020 ▸).

**Figure 2:**
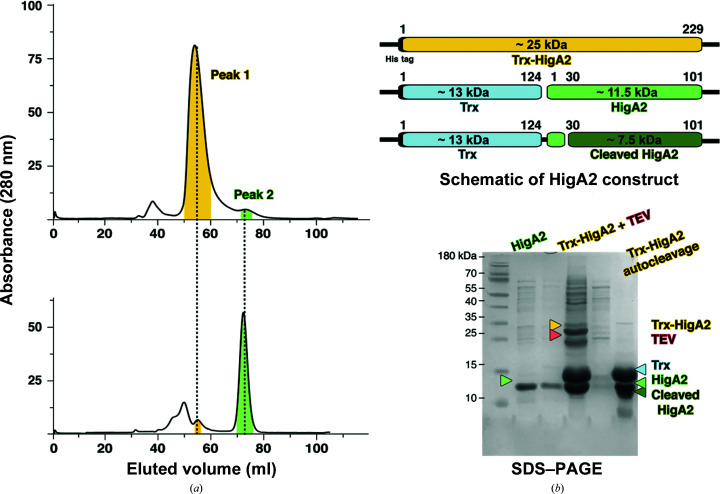
Autocleavage of _Mt_HigA2. (*a*) Size-exclusion chromatography of _Mt_HigA2 before (top) and after (bottom) TEV cleavage. The major peak coloured yellow reduces substantially, and a small peak shown in green became the major peak after purification. (*b*) Schematic diagram of _Mt_HigA2 and the corresponding SDS–PAGE. The colours correspond to the components in the _Mt_HigA2 construct.

**Figure 3:**
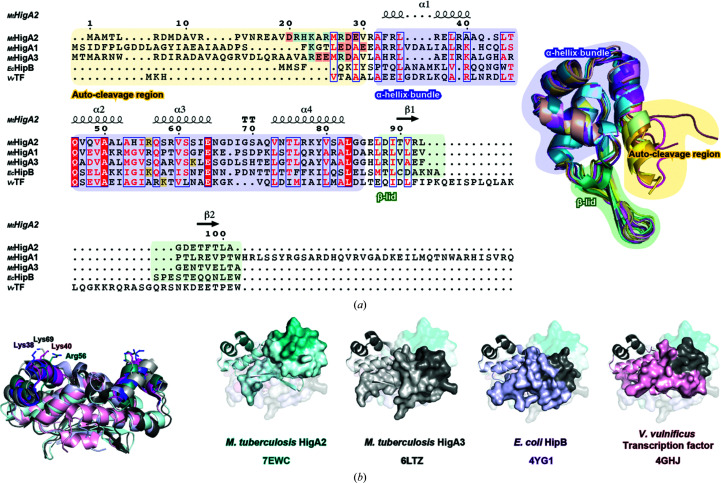
Sequence and structure comparison of _Mt_HigA2. (*a*) Sequence comparison of _Mt_HigA2 with the related proteins _Mt_HigA3 (40% sequence identity), _Mt_HigA1 (28% sequence identity), *E. coli* HipB (19% sequence identity) and *V. vulnificus* transcription factor (22% sequence identity). Three parts of _Mt_HigA2 (the N-terminal autocleavage region, α-helix bundle and C-terminal β-lid) are coloured with yellow, blue and green backgrounds. The four monomers from form I, the four monomers from form II and the five monomers from form III are aligned in the right panel. Each monomer shows a different state of the N-terminal residues, which implies that this region is intrinsically unstable and disordered. The charged _Mt_HigA N-terminal residues are coloured with light red and light blue backgrounds. The key amino acids for DNA interaction in *E. coli* HipB and _Mt_HigA3 are highlighted with a yellow background and the corresponding positive residues are coloured in the same way. The figure was constructed using *ESPript* (Robert & Gouet, 2014 ▸). (*b*) Superposition of _Mt_HigA2 with the structurally known proteins in (*a*). The superposition shows a similar fold, showing a conserved orientation of positively charged amino acids, which are coloured yellow in (*a*). However, when they are aligned based on HTH motifs, a unique linear coordination of _Mt_HigA2 is observed. PDB codes are shown below the structures.

Since the autocleavage of _Mt_HigA2 has been confirmed to be biologically important, size-exclusion chromatography (SEC) was employed to determine when the protein is cleaved. As _Mt_HigA2 lacks tryptophan, it possesses a low molar extinction coefficient (1490 *M* ^−1^ cm^−1^), but it is still detectable due to a single tyrosine (Tyr79) [Fig. 2 ▸(*a*)]. However, the Trx-_Mt_HigA2 construct showed an indicative absorbance level since it has an additional N-terminal Trx tag. Cleavage using TEV protease results in Trx (13 kDa) and _Mt_HigA2 (11.5 kDa), and a similar result is observed when the protein is preserved at 4–20°C overnight without TEV protease. In both cases, _Mt_HigA2 is cleaved further within hours [Fig. 2 ▸(*b*)]. The predicted cleavage site resides between residues 20 and 29, where a significant number of charged residues are present (eight out of ten) [Fig. 3 ▸(*a*)]. Disorder analysis with *DisEMBL* supports the observation in the crystal structure that the N-terminus is intrinsically disordered and is spontaneously cleaved in the absence of the toxin (Linding *et al.*, 2003 ▸).

### 3.3. Comparison of *M. tuberculosis* HigA2 with related proteins   

A total of three _Mt_HigBA pairs have been identified in *M. tuberculosis* H37Rv, including HigBAC and HigBA1. In the present study, we determined the structure of _Mt_HigA2 and compared it with the previously elucidated _Mt_HigA3 structure (Eickhoff *et al.*, 2009 ▸). Both share the same secondary-structure topology (α1–α2–α3–α4–β1–β2), with an apparently similar fold, but show a high backbone r.m.s.d. and a low *DALI* *Z*-score (2.6 Å and 8.4, respectively). The *DALI* server reveals other strong matches in type II TA systems: *E. coli* HipB and *V. vulnificus* transcription factor. Despite low structural sequence identity, they all share a HTH motif in each monomer, but the β-lid is only preserved in *E. coli* HipB and _Mt_HigA3.

When the structures and sequences are aligned, the unique characteristics of _Mt_HigA2 are revealed. The first notable difference between the _Mt_HigA proteins and the other two proteins lies in the N-terminus. There are no corresponding sequences for this region in *E. coli* HipB and *V. vulnificus* transcription factor. Since the N-terminal residues of _Mt_HigA2 and _Mt_HigA3 are cleaved readily, the N-terminus of _Mt_HigA1 is also expected to be cleaved. The sequence homology is poor in the N-terminal region but the expected cleavage region shows similarity, with prominent charged residues. Therefore, in the absence of the toxin the N-terminus of _Mt_HigA adopts an intrinsically disordered form that is prone to spontaneous autocleavage. A similar characteristic is detected in *V. cholerae* HigA, showing the convergence of charged residues at the N-terminal expected cleavage site. Another distinctive feature is found in the HTH motif, which is responsible for nucleic acid binding. Previous structural studies of _Mt_HigA3 and *E. coli* HipB specified the DNA-interacting residues, which are predominantly positively charged amino acids such as Lys69 in _Mt_HigA3 and Lys38 in *E. coli* HipB. The side chains of both residues protrude to the surface to interact with the negatively charged phosphate or bases of DNA. When superimposed, all four structures show positively charged residues in each corresponding region, Lys40 in *V. vulnificus* transcription factor and Arg56 in _Mt_HigA2, implying charge conservation for DNA binding. However, low sequence identity in the HTH motif is likely to influence sequence specificity. The last characteristic feature is found in the C-terminus, with _Mt_HigA1 having an additional ∼40 residues. Aside from the typical toxin–antitoxin systems _Mt_HigBA2 and _Mt_HigBA3, _Mt_HigBA1 is a member of a toxin–antitoxin–chaperone system. Rv1955, Rv1956 and Rv1957 perform these functions as HigB1 (toxin), HigA1 (antitoxin) and chaperone, respectively (Sala *et al.*, 2014 ▸). The crystal structure of the chaperone, Rv1957, was determined with the C-terminal peptide of _Mt_HigA1 (Rv1956), implying that the additional C-terminal residues of _Mt_HigA1 (Rv1956) may play a key role in stable complex formation with the chaperone (Guillet *et al.*, 2019 ▸). _Mt_HigA2 and _Mt_HigA3 lack C-terminal residues and do not possess a third gene in their respective operons, highlighting that an additional chaperone protein is not required for folding (Fig. 3 ▸).

### 3.4. The unique characteristics of *M. tuberculosis* HigA2   

The human pathogen *M. tuberculosis* H37Rv contains 79 toxin–antitoxin pairs, whilst other mycobacteria possess fewer, implying a clinically relevant correlation. Of these 79, 38 are classified as type II, which includes the HigBA system. The canonical hierarchy for TA systems has the antitoxin gene located upstream of the toxin, likely as a regulatory control, but in the HigBA system this gene order is swapped. It is not known why the HigB toxin gene is the first gene of the operon, although the antitoxin does not possess its own promoter (Armalytė *et al.*, 2018 ▸; Park *et al.*, 2020 ▸). However, unlike the previous study on _Mt_HigBA3, the promoter region for _Mt_HigBA2 was not clearly defined. The *BPROM* tool failed to identify the σ_70_ binding site (Solovyev & Salamov, 2011 ▸). Therefore, four DNA sequences around the −10 box and the −35 box of _Mt_HigA2 and _Mt_HigBA2 were used in an inter­action assay using EMSA, ITC and SEC, as in our previous study on _Mt_HigA3. However, binding was not detected for _Mt_HigA2 (Supplementary Fig. S2).

Although the _Mt_HigA2 dimer and _Mt_HigA3 dimer showed similarities in three-dimensional structures (backbone r.m.s.d. of 2.6 Å), _Mt_HigA2 shows a major difference in the arrangement of the two HTH motifs from each monomer. _Mt_HigA3 showed an arched formation of HTH motifs, while _Mt_HigA2 has a linear arrangement (Park *et al.*, 2020 ▸). This linear configuration is unique compared with similar transcription factors [Fig. 3 ▸(*b*)]. From our study, the linear arrangement and negative result in DNA binding suggest that a rearrangement of the HTH motif is required for interaction with promoter DNA.

### 3.5. Suggested model for interaction of *M. tuberculosis* HigA2 with DNA or HigB2   

**Figure 4:**
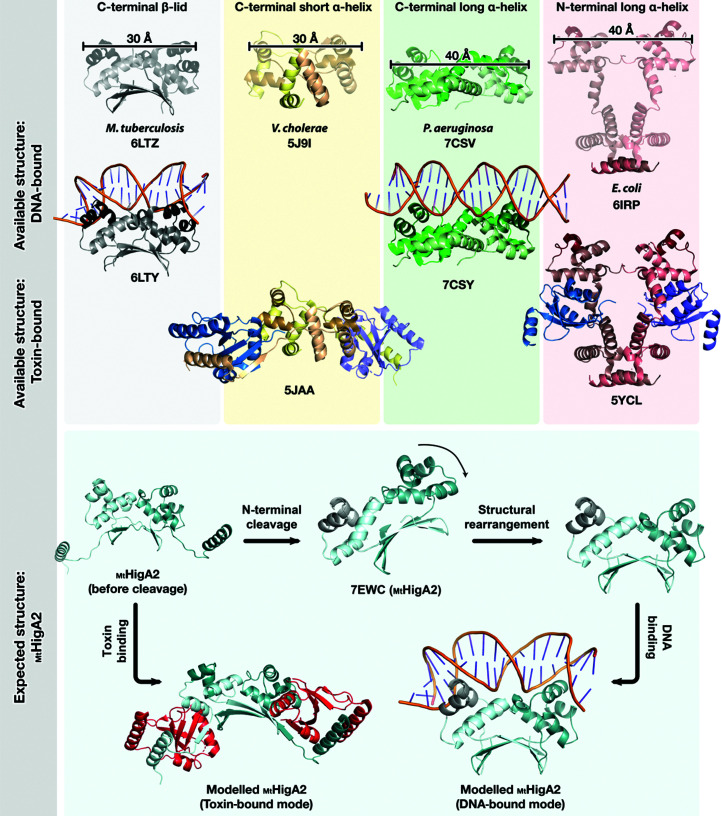
Expected binding mode of _Mt_HigA2. HigA shows diverse dimerization modes. The major contributor to dimerization is highlighted and HTH motifs are coloured in a darker shade. The distance between two HTH motifs is shown in Å. The DNA-bound structures are available for *M. tuberculosis* and *P. aeruginosa* and toxin–antitoxin complex structures are available for *V. cholerae* and *E. coli* HigA. From the comparison, we could model the DNA-binding mode and toxin-binding mode of _Mt_HigA2 (coloured in cyan). PDB codes are shown below the structures.

The common structural characteristic of dimeric HigA antitoxins is that they bind to a specific promoter DNA using HTH motifs from each monomer. However, the HigA HTH motifs show various arrangements as a consequence of dimerization. While _Mt_HigA2 and _Mt_HigA3 use a C-terminal β-lid for dimerization, *P. aeruginosa* HigA and *P. vulgaris* HigA utilize a long α-helix and *V. cholerae* HigA uses a short α-helix at the C-terminus for dimerization (Song *et al.*, 2021 ▸; Schureck *et al.*, 2019 ▸; Hadži *et al.*, 2017 ▸). Another dimerization mode is found in *E. coli* HigA and *Shigella flexneri* HigA, showing close contact through the N-terminal α-helix (Fig. 4 ▸). The dimerization determines the position of the HTH motifs, which recognize DNA major grooves as pairs. When the dimer is formed by a C-terminal long α-helix or an N-terminal α-helix, the distance between each HTH motif is ∼40 Å, whilst it is ∼30 Å for dimers that use a β-lid or a C-terminal short α-helix. Although the number of antitoxin–DNA complexes in the PDB is low, the DNA-bending function is predictable from the distances between the two HTH motifs.

To identify the binding mode of _Mt_HigA2 to DNA, we performed docking simulations to the corresponding DNA with _Mt_HigA2 in linear and bent conformations using *ClusPro* (Desta *et al.*, 2020 ▸). The linear _Mt_HigA2 model was chosen from our highest resolution structure (PDB entry 7ewc) and the bent conformation was modelled using _Mt_HigA3. For docking, the DNA was defined as the receptor, with _Mt_HigA2 as the ligand. Among 7000 rotations, the top ten lowest scoring results were identified and visually inspected in *PyMOL*. Docking trials failed when the linear _Mt_HigA2 was docked to linear DNA, which agrees with our experimental observations (EMSA, ITC and SEC). Docking highlights that _Mt_HigA2 binds to both bent and linear DNA upon structural rearrangement. Without the rearrangement of _Mt_HigA2, docking shows a disfavoured interaction with DNA as the HTH motifs remain exposed to the solvent area. When bent _Mt_HigA2 interacts with linear DNA, the distance between the HTH motifs is widened by a β-lid distortion (Supplementary Fig. S3). The docking experiments clearly indicate that structural rearrangement is necessary for interaction with DNA (Supplementary Fig. S3). We can also predict the _Mt_HigA2–_Mt_HigB2 complex structure from *V. cholerae* HigA, because a cleavable N-terminus is surmised to be involved in the formation of a toxin–antitoxin complex. The structural diversity found in _Mt_HigA2 requires flexible dimerization, which is closely related to interaction with the DNA or toxin.

## 4. Discussion   

Structural studies of _Mt_HigA2 confirmed that it has three regions: (i) a disordered N-terminus that is liable to cleavage, (ii) an α-helix bundle containing an HTH motif for DNA binding and (iii) a β-lid for dimerization. The N-terminal part is known to be responsible for interaction with the toxin, but when the antitoxin is solely expressed this part spontaneously autocleaves, showing TA-system regulation at the protein level. In the absence of the N-terminal part, _Mt_HigA2 forms a stable dimer both in solution and in the crystal structure on account of the extensive hydrogen bonds observed in the β-lid. The three different crystal structures in our study reveal the flexibility in dimerization mode while anchored by the β-lid.

**Figure 5:**
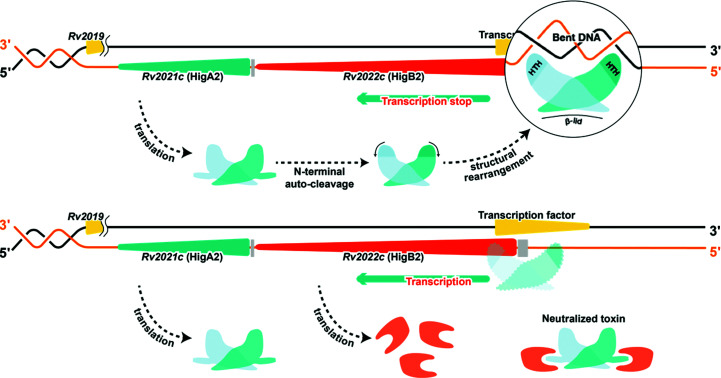
_Mt_HigBA2 operon and action of _Mt_HigA2. Based on our results, a schematic of the _Mt_HigBA system is suggested. _Mt_HigA2 is translated downstream of _Mt_HigB2. Upon translation, the N-terminus of _Mt_HigA2 is autocleaved and structural rearrangement occurs to bind DNA, thereby blocking additional expression of the HigBA operon. When the _Mt_HigB toxin is expressed, _Mt_HigA2 with an intact N-terminus binds to the toxin and neutralizes it.

Antitoxins function to neutralize toxin activity either by direct toxin binding or by repression of expression (negative autoregulation). The antitoxin _Mt_HigA2 has two binding partners: DNA and the toxin _Mt_HigB2. Although _Mt_HigA2 was expected to bind DNA using HTH motifs, we obtained negative results in interaction studies. To fit two HTH motifs to the DNA major grooves, the structure should cover the length between two major grooves, which is 34 Å. However, the distance between two HTH motifs in _Mt_HigA2 is 30 Å, meaning that DNA bending is indispensable. Despite this, the HTH motifs in _Mt_HigA2 arrange linearly, implying that structural rearrangement is required for DNA binding. Upon DNA binding, the expression of the toxin–antitoxin operon is downregulated. Another binding partner is the _Mt_HigB2 toxin. The N-terminal antitoxin has an autocleavable ∼25 residues and this part is predicted to bind the C-terminus of the toxin. When _Mt_HigB2 is translated because of weak _Mt_HigA2–DNA binding, the toxin action can still be blocked by the formation of an _Mt_HigBA complex with successively translated _Mt_HigA2. The toxin _Mt_HigB2 inhibits protein synthesis by mRNA cleavage and causes cell-growth arrest and cell death. Therefore, the antitoxin _Mt_HigA2 has a double protection system to neutralize the toxin (Fig. 5 ▸). Further studies of DNA- or toxin-bound structures remain to be performed. However, this study contributes various methods by which the antitoxin may control its function, which include autocleavage or structural flexibility. This will provide useful information for future studies of gene-regulating proteins.

## Supplementary Material

PDB reference: *Mycobacterium tuberculosis* HigA2, 7ewc

PDB reference: 7ewd

PDB reference: 7ewe

Supplementary Figures. DOI: 10.1107/S2052252521007715/lz5052sup1.pdf

## Figures and Tables

**Table 1:** Crystal data-collection and refinement statistics Values in parentheses are for the highest resolution shell.

	Form I	Form II	Form III
Data collection			
Beamline	BL-5C, PLS	BL-7A, PLS	I04, DLS
Wavelength (Å)	0.98	0.98	0.98
Resolution range (Å)	40.0–2.1	40.0–3.2	77.0–3.4
Space group	*P*2_1_2_1_2_1_	*P*4_3_2_1_2	*P*3_1_21
*a*, *b*, *c* (Å)	30.610, 89.955, 114.961	67.621, 67.621, 190.646	80.890, 80.890, 153.910
α, β, γ (°)	90, 90, 90	90, 90, 90	90, 90, 90
Observations (total/unique)	379872/20447	14009/7877	47017/9603
Completeness (%)	99.8 (99.7)	100 (100)	99.5 (99.2)
*R*_merge_ †	0.079 (0.261)	0.105 (0.757)	0.112 (2.663)
CC_1/2_	0.99 (0.98)	0.98 (0.89)	0.99 (0.51)
Multiplicity	6.0 (6.2)	7.4 (7.4)	4.9 (5.1)
〈*I*/σ(*I*)〉	29.0 (15.5)	24.7 (7.0)	7.9 (0.9)
Refinement			
*R*_work_/*R*_free_ ‡ (%)	20.9/24.7	21.5/26.4	20.6/27.7
Average *B* value (Å^2^)	35.4	99.6	126.4
R.m.s.d., bond lengths (Å)	0.010	0.009	0.009
R.m.s.d., angles (°)	1.693	1.697	1.849
Ramachandran plot (%)			
Favoured	98.88	89.32	82.74
Allowed	1.12	7.83	13.74
Disallowed	0	2.85	4.09

† *R*_merge_ = \textstyle \sum_{hkl}\sum_{i}|I_{i}(hkl)- \langle I(hkl)\rangle|/\textstyle \sum_{hkl}\sum_{i}I_{i}(hkl), where *I_i_* (*hkl*) is the *i*th measured intensity of reflection *hkl* and 〈*I*(*hkl*)〉 is the mean of all measured intensities of reflection *hkl*.

‡ *R*_work_ = \textstyle \sum_{hkl}\big ||F_{\rm obs}|-|F_{\rm calc}|\big |/ \textstyle \sum_{hkl}|F_{\rm obs}|, where *F*_obs_ is the observed structure-factor amplitude and *F*_calc_ is the structure factor calculated from the model. *R*_free_ is computed in the same manner as *R*_work_ but from a test set containing 5% of the data, which were excluded from the refinement calculation.
