# Supplementary material for: Chasing the structural diversity of the transcription regulator Mycobacterium tuberculosis HigA2
Source: IUCrJ. 2021 Aug 24;8(Pt 5):823–32. doi: 10.1107/S2052252521007715 (PMC8420761; doi:10.1107/S2052252521007715)
Supplement: Supplementary file 1 [file m-08-00823-sup1.pdf]

IUCrJ

Volume 8 (2021)

Supporting information for article:

Chasing the structural diversity of the transcription regulator *Mycobacterium tuberculosis* HigA2

William Richardson, Gyun Won Kang, Hee Joong Lee, Kang Mu Kwon, Saron Kim and Hyo Jung Kim

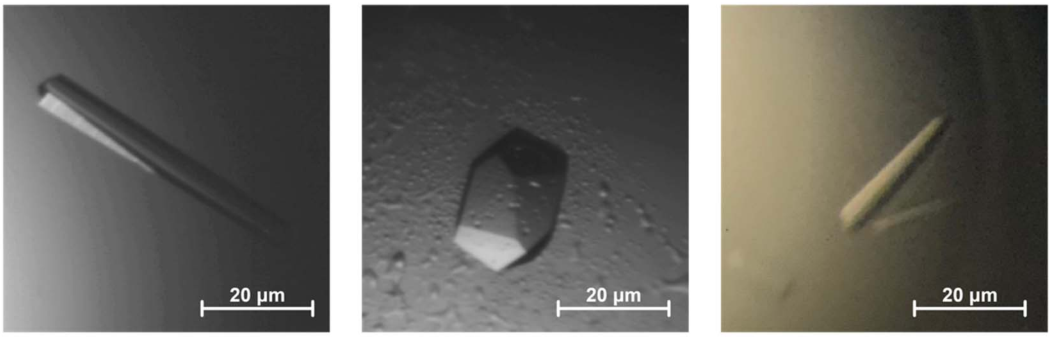

Form I Form II Form III

Figure S1 Morphology of $_{Mt}$HigA2 crystals. Three protein crystals differ from each other in crystal shapes and sizes.

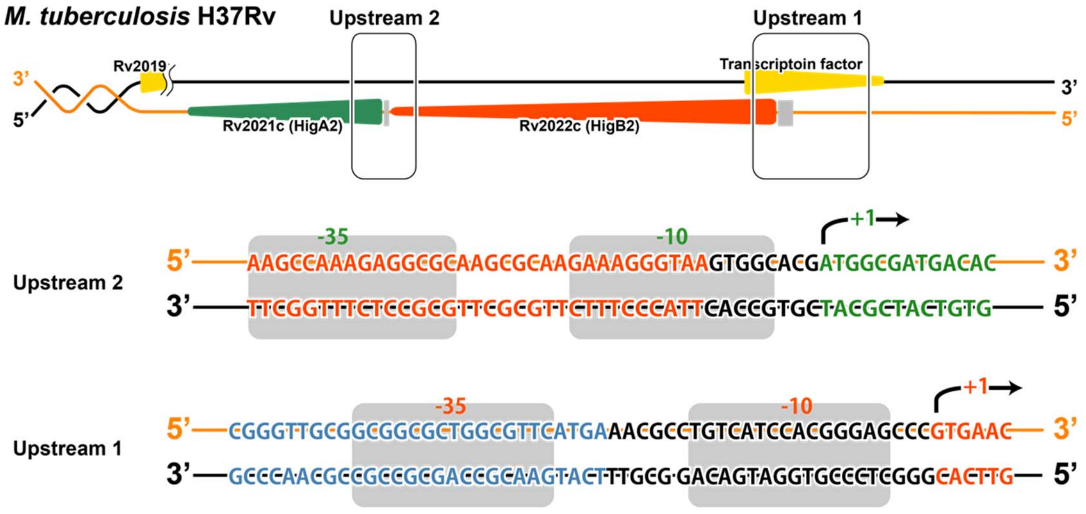

Figure S2 Organization of $_{Mt}$HigBA2 operon and expected promoter sequence. The putative -10 box and -35 box are denoted. The DNA sequence tried for interaction assay were highlighted as grey colour.

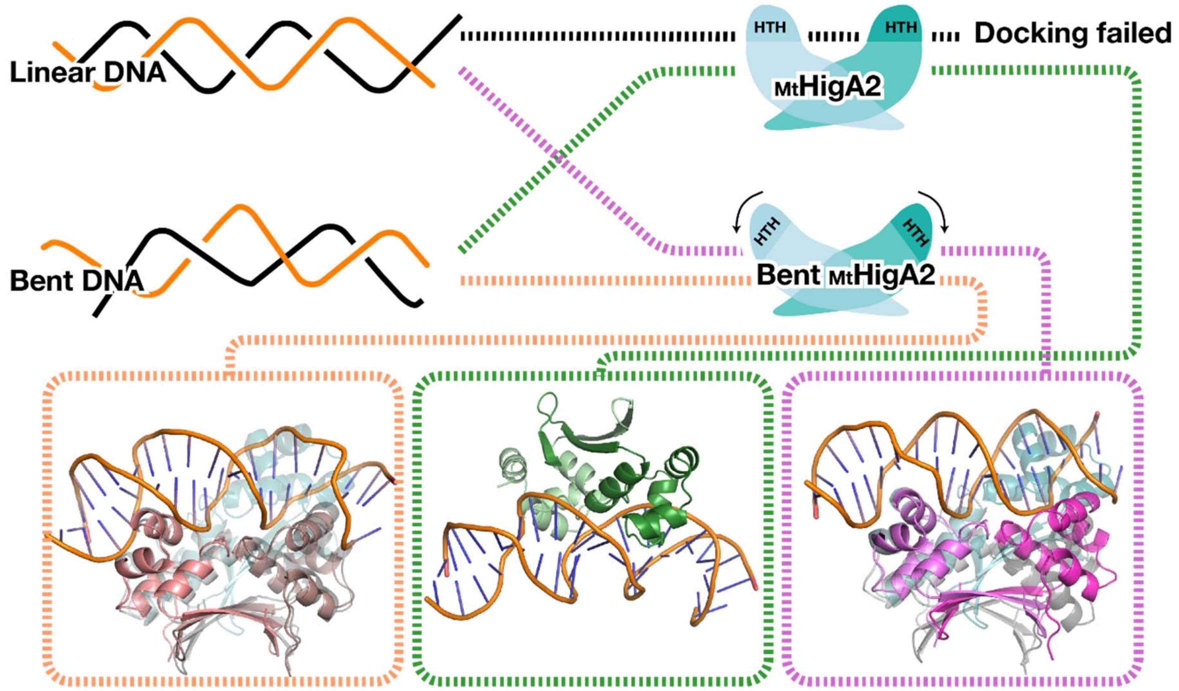

Figure S3 Proposed interaction between the MtHigA2 and DNA. Total 4 sets of docking simulation were tried but linear MtHigA2 failed to recognize linear DNA. The lowest scored DNA-bound model was identified when bent MtHigA2 was docked to bent DNA (coloured in salmon, weighted ClusPro score of -2103). When the linear MtHigA2 is docked to bent DNA, the wrong binding model was resulted, showing solvent-exposed HTH motifs (coloured in green, weighted ClusPro score of -1595). Linear MtHigA2 was successfully docked to bent DNA, but distorted β-lid is observed in docked model (coloured in magenta, weighted ClusPro score of -2003). Linear MtHigA2 (light cyan) and modelled bent MtHigA2 (light grey) are aligned based on HTH motif and superimposed.
